# Variability in Assembly of Degradation Operons for Naphthalene and its derivative, Carbaryl, Suggests Mobilization through Horizontal Gene Transfer

**DOI:** 10.3390/genes10080569

**Published:** 2019-07-27

**Authors:** Prashant S. Phale, Bhavik A. Shah, Harshit Malhotra

**Affiliations:** Department of Biosciences and Bioengineering, Indian Institute of Technology-Bombay, Powai, Mumbai 400 076, India

**Keywords:** xenobiotics, naphthalene, Carbaryl, horizontal gene transfer, mobile genetic elements, transposons, integrative conjugative elements, pathway assembly, evolution

## Abstract

In the biosphere, the largest biological laboratory, increased anthropogenic activities have led microbes to evolve and adapt to the changes occurring in the environment. Compounds, specifically xenobiotics, released due to such activities persist in nature and undergo bio-magnification in the food web. Some of these compounds act as potent endocrine disrupters, mutagens or carcinogens, and therefore their removal from the environment is essential. Due to their persistence, microbial communities have evolved to metabolize them partially or completely. Diverse biochemical pathways have evolved or been assembled by exchange of genetic material (horizontal gene transfer) through various mobile genetic elements like conjugative and non-conjugative plasmids, transposons, phages and prophages, genomic islands and integrative conjugative elements. These elements provide an unlimited opportunity for genetic material to be exchanged across various genera, thus accelerating the evolution of a new xenobiotic degrading phenotype. In this article, we illustrate examples of the assembly of metabolic pathways involved in the degradation of naphthalene and its derivative, Carbaryl, which are speculated to have evolved or adapted through the above-mentioned processes.

## 1. Introduction

Anthropogenic activities like manufacturing, industrialization and combustion of fossil fuels have led to the release of a large number of compounds in the environment, which were previously unknown to the biosphere. These compounds, known as xenobiotics, are toxic to various life forms and persist in the environment for a long period of time. Monocyclic and polycyclic aromatic hydrocarbons (PAHs) form a major group of xenobiotics. Due to the highly reduced nature, resonance stabilized structure, greater hydrophobicity, and lower aqueous solubility, they are resistant to biodegradation (recalcitrant). They act as metabolic inhibitors, endocrine disrupters [1] and further reactive epoxide formed due to metabolic activation of these compounds by microsomal enzymes interacts with DNA, causing mutation(s) or chromosomal damage [2,3,4] which may lead to cytotoxicity, genotoxicity and/or carcinogenicity [5]. Repeated application and release of these compounds in the environment has exerted a selection pressure onto the microbes, resulting in the evolution of novel/unique degradative pathways. Although processes such as volatilization, chemical oxidation and photo-oxidation contribute to degradation, bioremediation is the most efficient and cost-effective method for complete removal of these compounds [6,7]. In this review, we focus on the metabolism and various genetic elements involved in the assembly of the degradative pathways for naphthalene and its derivative, Carbaryl.

Naphthalene is ubiquitous in the environment and used extensively in the manufacture of various compounds including pesticides and plastics, as well as for domestic consumption (mothballs, insect repellent). Being hydrophobic, it partitions into the membranes [8] and gets accumulated in tissues of aquatic organisms, leading to toxicity [9,10]. In humans, exposure to naphthalene occurs via skin, oral ingestion or inhalation of fumes. Acute toxicity causes hemolytic anemia [11], whereas exposure to higher doses causes cerebral oedema and chronic renal failure [12]. Naphthalene has been classified as a potential human carcinogen based on the studies performed on experimental animals [13].

Carbaryl (1-naphthyl *N*-methylcarbamate), a derivative of naphthalene, is a broad-spectrum carbamate family insecticide that has been manufactured and used since 1960. It acts as a competitive inhibitor of the enzyme acetylcholine esterase of the central nervous system leading to paralysis [14]. It is highly toxic to aquatic invertebrates, amphibians, bees, earthworms and humans [15,16,17,18,19,20,21]. It has also been classified as a likely human carcinogen based on vascular tumour formation in mice [22]. In nature, various bacterial species belonging to genus *Pseudomonas, Rhodococcus, Mycobacterium, Nocardia*, *Bacillus*, Vibrio, Marinobacter**, *Micrococcus* and *Sphingomonas* have been reported to degrade naphthalene and Carbaryl.

## 2. Microbial Adaptation to Aromatics and Xenobiotics

Aromatics including PAHs and xenobiotics like pesticides, being toxic and persistent, act as a selection force on the microbial community to evolve degradative pathways and possibly remove sensitive microorganisms from the population [23,24,25]. Microbes adapt to these environmental challenges and acquire the ability to degrade these compounds using the following strategies:

a) Quite a few bacterial isolates have the ability to completely degrade and utilize mono as well as polycyclic aromatics like benzoate, phthalate isomers and their esters, chlorobenzoate, naphthalene, biphenyls, etc. [23,26,27,28,29,30,31,32,33,34,35,36,37,38,39,40,41]. Such organisms harbor genes encoding all enzymes responsible for the complete metabolism of these compounds as a sole source of carbon and energy. Further, these genes could be arranged as operon(s) so as to finetune their expression to achieve optimum degradation efficiency. In a few organisms, both the carbon source as well as detoxification pathways are functional. For example, in *Pseudomonas putida* CSV86, 1-methylnaphthalene, which is highly toxic and used as insect repellent, is metabolized by two pathways. It is transformed and detoxified by side chain hydroxylation pathway to 1-naphthoic acid, a less toxic and more water-soluble metabolite, which is released into the medium as a dead-end product [29]. Whereas in other pathway, 1-methylnaphthalene is ring-hydroxylated and further metabolized to central carbon pathway intermediates, thus acting as sole source of carbon and energy [42].

b) In biosphere, microbial communities degrade a diverse range of pollutants. Also, specific consortia are constructed in the laboratory based on their metabolic properties and compatibility of organisms to degrade the pollutants. Members of such communities/consortia achieve complete mineralization by working together, which is mutually beneficial. Initial transformation of the parent compound by one member yields metabolite(s) which may be a dead-end product(s) for its metabolic machinery, which can then be subsequently used by other member(s). To list a few examples: consortia of *Pseudomonas* spp. 50552 and 50581 involved in complete degradation of Carbaryl [43]; EC20 consortia of *Pseudomonas, Mesorhizobium, Achromobacter, Stenotrophomonas,* and *Halomonas* involved in BTEX (benzene, toluene, ethylbenzene and xylene) degradation [44]; consortia of five fungal and eight bacterial isolates to degrade phenanthrene, pyrene, and benzo(a)pyrene [45]; ASDC consortia of *Rhodococcus* sp., *Bacillus* sp., and *Burkholderia* sp. degrading chrysene [46]. Several bacteria, algae and fungi have been reported to transform methylnaphthalene into naphthoic acid [47,48] which can then be metabolized by other microbes like *Stenotrophomonas maltophilia* CSV89 [49].

c) Efficient degradation of certain xenobiotics can be achieved by supplementing with specific nutrient(s). For example, isophthalate, which is used in the plastic and textile industry, acts as a competitive inhibitor for the enzyme glutamate dehydrogenase (GDH), involved in the C/N metabolism. When the growth medium is supplemented with glutamate, GDH is relieved from the competitive inhibition by isophthalate, thereby enhancing the degradation efficiency of isophthalate in *Pseudomonas* sp. [50]. On the other hand, soil isolate *Acinetobacter lwofii* ISP4 degrades isophthalate faster and more efficiently without any supplementation of glutamate by expressing GDH, which has less sensitivity to inhibition by isophthalate and is synthesized in more quantity [51]. These examples indicate the adaptation by the microbes at the metabolic level for their effective survival.

## 3. Horizontal Gene Transfer Elements Involved in Catabolism of Aromatics

Genetic variation in microorganisms is the major driving factor in adaptation to environmental conditions. The metabolic diversity observed in the organism(s) can be attributed to the plasticity of the genome which is acquired through various strategies, resulting in the generation of genetic variants and ultimately adaptation, as described by Arber [52]. These include: (i) small local change(s) in the nucleotide sequence, (ii) reshuffling of gene fragments within the genome, and/or iii) horizontal gene transfer (HGT). The third strategy allows for evolution in quantum leaps [53] and is mediated through mobile genetic elements (MGEs) like plasmids (conjugative and non-conjugative), transposons, integrative conjugative elements (ICEs) and genomic islands (GEIs), as well as phages and prophages. The association of MGEs with catabolic gene clusters, as well as high levels of similarity in gene organization and nucleotide sequences among phylogenetically and geographically distant microbial species suggest that HGT is the major player in acquisition and assembly of the degradative pathways in microorganisms [25,54,55,56,57]. The presence of PAH-degradation genes on MGEs is an indication of ease of mobilization of catabolic genes and hence better adaptability to the polluted environment [58].

Catabolic plasmids involved in the degradation of camphor, octane, naphthalene, salicylate, etc. were first discovered in the early 70s [59,60,61,62,63]. These plasmids can either be conjugative or non-conjugative in nature. Plasmid isolation and characterization, and plasmid curing by chemicals as well as by nutritional conditions, southern hybridization, conjugation and transformation experiments established the association of the degradative property to the plasmid(s) [64,65,66,67]. Advances in genomic techniques and approaches have further provided insights into the evolutionary aspects of degradation properties [68,69,70]. Presence of insertion elements harboring functional or non-functional transposase and integrase like features have been seen to be associated with degradative genes in large number of catabolic plasmids like pWW0 and NAH7 amongst others [71,72]. The ability of some of these plasmids to move across various genera and strains make them carriers of the degradative property. Plasmids play an important role in the evolution of metabolic diversity in Pseudomonads [73].

Catabolic transposons form the major group of MGEs involved in the transfer of xenobiotic degradation property [74,75,76]. Transposons can be grouped into three classes based on the nucleotide sequence homology, genetic organization and mechanistic properties [77]. Class-I elements include composite transposons and simple insertion sequences (ISs). Simple ISs only contain the elements essential for transposition and are flanked by inverted or direct repeats (DRs). Composite transposons have genes encoding features other than transposition and are flanked by very similar ISs in direct or inverted position. For example, catabolic transposon Tn*5280,* harboring genes encoding chlorobenzene dioxygenase and dehydrogenase, was found to be part of the plasmid pP51 in *Pseudomonas* sp. strain P51 [78]. Class II transposons, known to transpose by the formation of co-integrate, are known to carry gene fragments greater than 50 kb. Transposon Tn*4651* which harbors the *xyl* operon*,* is a part of Tn*4653* and is located on the plasmid pWW0 in *P. putida* mt-2 [71,79]. The third class of transposons is called as conjugative transposons. 

Elements like ICEs refer to a group of MGEs which had earlier been classified under different groups like conjugative transposons, integrative plasmids and GEIs [25,80]. These elements are excised by site-specific recombination yielding circular intermediates which are then transferred by conjugation to other bacteria and can possibly re-integrate into the genome [25,80]. Elements like GEIs have been described as MGEs residing on the genome in close proximity to a gene for a tRNA and harbor a gene for integrase and/along with a short duplication of the insertion element at the other end [53,54,81].

**Figure 1:**
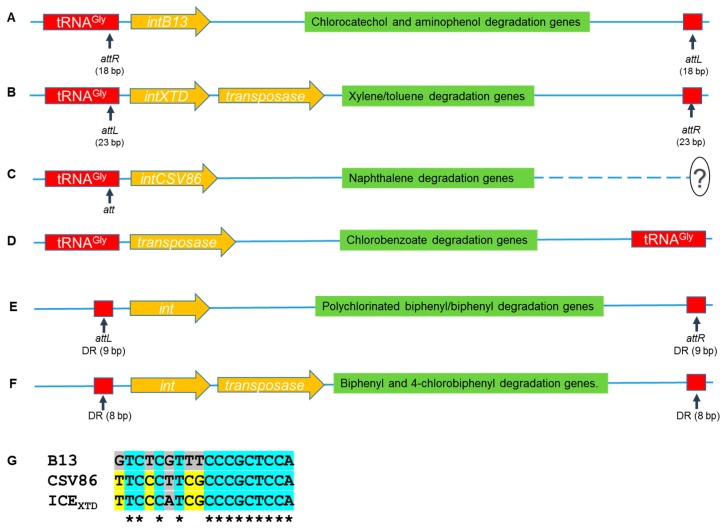
The structural features of various integrative conjugative elements (ICEs) involved in aromatic compound metabolism from: (**A**) *clc* element of *Pseudomonas* sp. B13; (**B**) ICE*_XTD_*of *Azoarcus* sp. CIB; (**C**) ICE*_CSV86_*element of *Pseudomonas putida* CSV86; (**D**) *clc* element of *Pseudomonas aeruginosa* JB2; (**E**) ICE*_KKS102_*4677 from *Acidovorax* sp. strain KKS102; and (**F**) Tn*4371* transposon most likely to be an ICE from *Cupriavidus oxalaticus*. Panel (**G**) represents multiple sequence alignment of attachment site (‘*att*’) region of the 3’ end of tRNA^Gly^ of *Pseudomonas* sp.B13, as B13, *Pseudomonas putida* CSV86 as CSV86 and *Azoarcus* sp. CIB as ICE*_XTD_*. Question mark in panel C depicts the incomplete/unidentified downstream region and *attR* site. Figure is not to the scale. tRNA: Transfer-RNA.

Pathogenicity islands (PAIs), a class of GEIs associated with the virulence phenotype, were first discovered in *E. coli* [82] and since then a wide plethora has been uncovered. Various disease associated toxins [83,84], antibiotic resistance genes [85] and even superantigens [86] are harbored on these MGEs. The similarity between the insertion sites of PAIs and phages at tRNA loci suggests that these islands are acquired through phage mediated HGT [87]. Also, certain PAIs harbor open reading frames that show high similarity to integrase of bacteriophages [88]. In comparison, conjugative transposons contain multiple integration sites and are not confined to tRNA genes. A few ICEs/GEIs are also reported to harbor genes involved in the degradation of various aromatic compounds; for example: *clc* element, for the metabolism of chlorobenzoate in *Pseudomonas* sp. strain B13 [89]; ICE*_XTD_* for *m*-xylene, toluene and cumene degradation in *Azoarcus* sp. CIB [90]; ICE*_CSV86_* for naphthalene degradation in *P. putida* CSV86 [91]; ICEclc JB2 for metabolism of *o*-halobenzoate and *o*-hydroxybenzoate in *P. aeroginosa* JB2 [92]; ICE_KKS102_4677 for the metabolism of polychlorinated biphenyl/biphenyl in *Acidovorax* sp. strain KKS102 [93]; proposed ICE harboring Tn*4371*, for the metabolism of biphenyl and 4-chlorobiphenyl [94]; and *phn* island for the metabolism of phenanthrene in *Delftia* sp. Cs1-4 [95]. The features of ICEs involved in aromatic compound degradation are depicted in Figure 1. 

The *clc* element is the first GEI, reported and characterized in detail from *Pseudomonas* sp. strain B13 and involved in the degradation of 3-chlorobenzoate [89]. ICE*clc_B13_*, ICE*_XTD_*and ICE*_CSV86_* are associated with tRNA^Gly^ having *att* site at the 3’ end of tRNA (72% identity among all three *att* and 94% among ICE*_XTD_* and ICE*_CSV86_*, Figure 1G). In *P. aeruginosa* JB2, the *att* site was found to be absent. The *phn* island involved in phenanthrene degradation from *Delftia* sp. Cs1-4 is structurally distinct from the *clc* family ICEs as it is not associated with the tRNA gene and has an SXT/R391 type mobilization system [95]. ICE_KKS102_4677 harbors *bph* operon and shows similar arrangement as observed in *clc* of B13 and ICE*_CSV86_*, consisting of direct repeats (9 bp, *attL* and *attR* region in ICE_KKS102_4677) with *attL* at the start, followed by integrase. Also, it has been found to be integrated with the chromosome and shows low conjugal transfer frequency as observed in the case of CSV86 [93,96]. Transposon Tn*4371* consists of transposase (*tnpA*) and phage-like integrase (*int*) genes [94]. Tn*4371* and ICE_KKS102_4677 are flanked by 8 bp and 9 bp direct repeats (55.6% identity), respectively, which are not associated with/in proximity to tRNA^Gly^.

## 4. Assembly of Naphthalene Degradation Pathway

**Figure 2:**
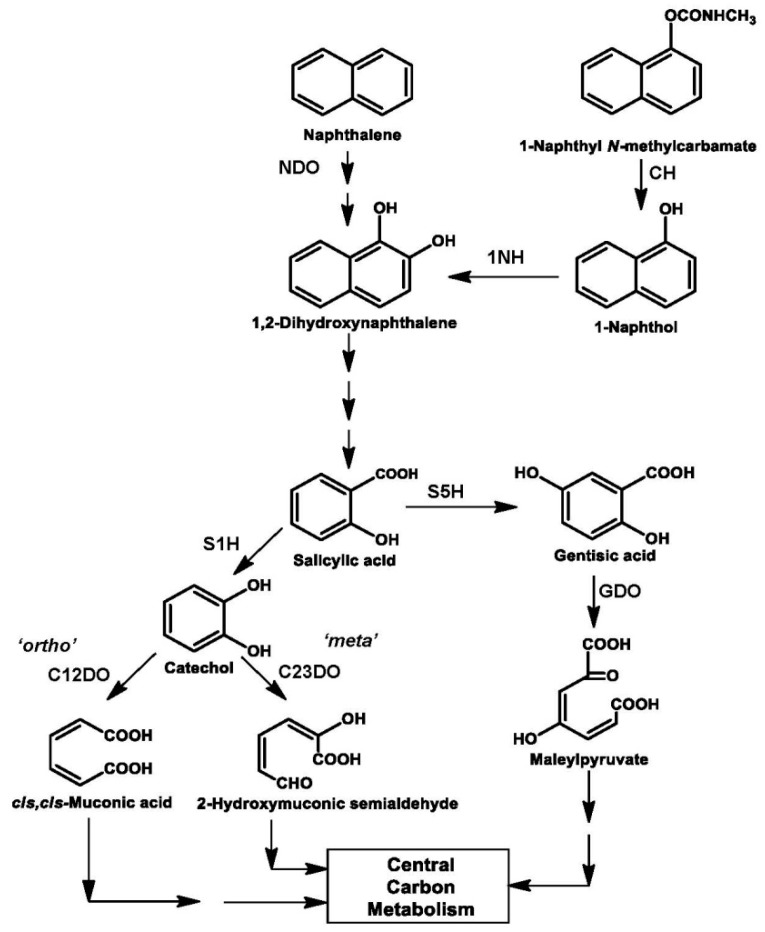
Metabolic diversity observed in naphthalene and Carbaryl degradation pathways from various soil bacterial isolates. Abbreviations are: NDO, naphthalene 1,2-dioxygenase; CH, Carbaryl hydrolase; 1NH, 1-naphthol 2-hydroxylase; S1H, salicylate 1-monoocxygenase; S5H, salicylate 5-hydroxylase; C12DO, catechol 1,2-dioxygenase; C23DO, catechol 2,3-dioxygenase; GDO, gentisate 1,2-dioxygenase. *ortho* and *meta* pathway indicates the mode of aromatic ring-cleavage by these enzymes.

Naphthalene, the simplest PAH, is used as a model compound to understand aromatic degradation pathways, enzymes and genetics. The number of microbes has been reported to metabolize naphthalene (Table 1A). The degradation is initiated by hydroxylation of one of the aromatic rings to yield 1,2-dihydroxynaphthalene, which is subsequently metabolized to salicylic acid. Generated salicylic acid is metabolized either via catechol (*meta* or *ortho* ring-cleavage) or gentisic acid to yield central carbon pathway metabolites like organic acids (Figure 2, Table 1A). Apart from these well studied routes, isolate *Bacillus thermoleovorans* was reported to degrade naphthalene via phthalic acid [97]. Based on biochemical, enzyme induction and regulation studies, naphthalene degradation pathway is segmented into *upper pathway* (naphthalene to salicylate, *nah* operon) and *lower pathway* (salicylate to central carbon pathway either via catechol, *sal* operon or gentisate, *gen*/*sgp* operon).

**Figure 3:**
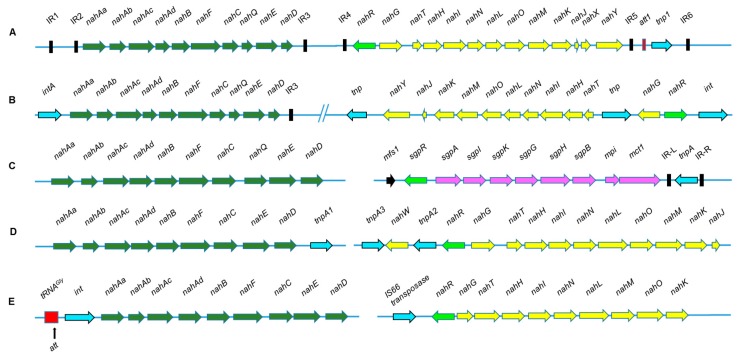
The diversity observed with respect to location, transcription and arrangement of genes involved in naphthalene degradation from: (**A**) *Pseudomonas putida* G7 as Tn*4655* transposon on plasmid NAH7 flanked by IR1 and IR6; (**B**) Pseudomonas *putida* strain NCIB9816-4 on conjugative plasmid pDTG1; (**C**) *Pseudomonas putida* AK5 on conjugative plasmid pAK5 via gentisate pathway; (**D**) *Pseudomonas stutzeri* AN10 on chromosome; (**E**) *Pseudomonas putida* CSV86 on ICE showing tRNA^Gly^ and integrase while the other end is still not known. Arrow indicates the direction of transcription. Genes responsible for upper pathway, ‘*nah’* operon (naphthalene to salicylic acid metabolism) are depicted in green; lower pathway, ‘*sal’* operon (salicylic acid to central carbon metabolites via catechol) are depicted in yellow; while those in pink are responsible for salicylate metabolism via gentisic acid, ‘*sgp*/*gen’* operon. Genes depicted in cyan arrows indicates transposase or integrase, black boxes depict IR elements, while red box indicates tRNA gene with ‘*att*’ site, light green arrow represents the regulators of the operons and the black arrow represents the putative integral membrane transport protein. Figure is not to the scale. Genes and proteins encoded are: *nahAa*, 2Fe-2S iron-sulfur cluster binding domain-containing protein, reductase; *nahAb*, non-heme iron oxygenase ferredoxin subunit; *nahAc,* naphthalene 1,2-dioxygenase; *nahAd*, naphthalene 1,2-dioxygenase subunit beta; *nahB*, *cis-*naphthalene dihydrodiol dehydrogenase; *nahF*, salicylaldehyde dehydrogenase; *nahC*, 1,2-dihydroxynaphthalene dioxygenase (oxygenase); *nahE*, 2-hydroxybenzalpyruvate aldolase; *nahD*, 2-hydroxychromene-2-carboxylate isomerase; *nahG*, salicylate 1-monooxygenase; *nahT,* 2Fe-2S iron-sulfur cluster binding domain-containing protein (chloroplast-type ferredoxin); *nahH*, catechol 2,3-dioxygenase; *nahI,* 2-hydroxymuconic semialdehyde dehydrogenase; *nahN*, 2-hydroxymuconic semialdehyde dehydrogenase; *nahL*, 2-oxopent 4-enoate hydratase; *nahO*, 4-hydoxy-2-oxovalerate aldolase; *nahM,* acetaldehyde dehydrogenase; *nahK,* 4-oxalocrotonate decarboxylase/2-oxo 3-hexendioate decarboxylase; *nahJ,* 4-oxalocrotonate tautomerase family protein; *nahY*, methyl accepting chemotaxis protein; *nahX*, unknown; *sgpA*, reductase component of salicylate 5-hydroxylase; *sgpI*, gentisate 1,2-dioxygenase; *sgpK*, fumarylacetoacetate hydrolase/fumarylpyruvate hydrolase; *sgpG*, salicylate 5-hydroxylase large oxygenase subunit; *sgpH*, salicylate 5-hydroxylase small oxygenase subunit; *sgpB*, ferredoxin component of salicylate 5-hydroxylase; *mpi,* maleylacetoacetate isomerase; *mct1*, methyl-accepting chemotaxis sensory transducer; *nahR* and *sgpR*, LysR family transcriptional regulator.

Metabolic diversity observed in naphthalene degradation pathways (Figure 2) is possible due to acquisition or exchange of genetic material. Genes for naphthalene degradation were found to be present on plasmid, chromosome, transposon or ICE (Table 1). The arrangement of genes encoding enzymes involved in naphthalene degradation is depicted in Figure 3. The degradation genes are arranged as two inducible operons: upper pathway (*nah*) and lower pathway (*sal* or *sgp/gen*) operon [98,99,100]. These operons are induced by salicylic acid and its analogue like 2-aminobenzoate and 2-hydroxybenzyl alcohol [100]. Among naphthalene degrading strains, the function of *nahR* was found to be highly conserved [101].

In *P. putida* G7, the plasmid NAH7 is known to harbor naphthalene degradation genes which are proposed to be part of a defective transposon which requires Tn*4653* transposase for mobilization [99]. In NAH7, the transcription for *nah* and *sal* operons is in the same direction. In *P. putida* strain NCIB9816-4, genes were found to be present on a conjugative plasmid pDTG1 as two operons (~15 kb apart) which are transcribed in opposite direction [102]. As compared to NAH7 and pDTG1, a non-conjugative pAK5 (IncP-7 group) from *P. putida* strain AK5 encodes naphthalene degradation via gentisate pathway as *nah* and *gen/sgp* operon [103]. In *P. putida* strain PMD-1, upper pathway genes (*nah* operon) were found to be located on the chromosome, while salicylate degradation genes (*sal* operon) were present on the conjugative plasmid pMWD-1 [104]. However, in *P. stutzeri* AN10, the naphthalene degradation genes (*nah* and *sal* operons) were found to be located on the chromosome and hypothesized to be recruited through transposition, recombination and rearrangement events [105,106]. Besides plasmids and chromosomes, naphthalene degradation genes were hypothesized to be present on ICE in *P. putida* CSV86 [91]. The draft genome analysis revealed that the *nah* operon is present next to tRNA*^Gly^*(along with *att* region) and phage-like integrase [91], suggesting the involvement of a GEI, referred to as ICE*_CSV86_*(Figure 3). This ICE is located on the chromosome and the degradation genes are arranged as *nah* and *sal* operons, which were transferred by conjugation at a very low frequency [96].

Interestingly, irrespective of the location of genes for naphthalene degradation either on plasmid, chromosome + plasmid, chromosome or ICE, the gene arrangement in *nah* and *sal* operon is almost conserved among various naphthalene degraders (Figure 3). Further, metabolic variations in isolates are generated through the regulation of operons and specificity of the enzyme(s), thus granting an advantage to the strain to metabolize naphthalene and other PAHs effectively [107,108].

## 5. Assembly of Carbaryl Degradation Pathway

Carbaryl has been used extensively in the agriculture sector since 1960. Repeated application has led microbes to adapt and utilize Carbaryl as the sole source of carbon and energy (Table 1B). The degradation pathway is initiated by hydrolyzing the ester bond by the enzyme Carbaryl hydrolase (CH) to yield 1-napthol. Generated 1-naphthol is ring-hydroxylated by the enzyme 1-naphthol hydroxylase (1NH) yielding 1,2-dihydroxynaphthalene, which is further metabolized via salicylic acid and gentisic acid. Few Carbaryl degraders also metabolize salicylic acid via catechol route [44] (Table 1B and Figure 2). The metabolic steps involved in the conversion of 1,2-dihydroxynaphthalene to salicylic acid are common for naphthalene and Carbaryl degradation (Figure 2). Interestingly, in naphthalene degraders salicylic acid is predominantly metabolized via catechol, whereas in Carbaryl degraders, it is metabolized mainly through the gentisic acid route (Table 1B and Figure 2).

**Figure 4:**
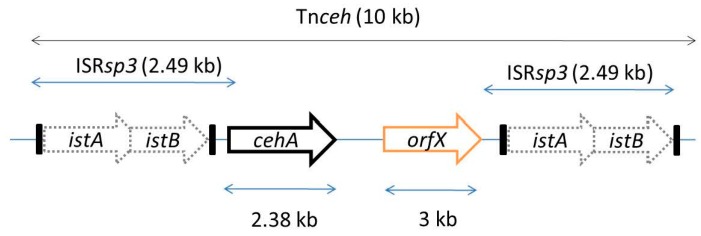
Organization of insertion elements observed in Carbaryl degrading *Rhizobium* sp. strain AC101 harboring gene *cehA* encoding Carbaryl hydrolase. Figure is not to scale.

A large number of genes involved in Carbaryl degradation have been proposed to be plasmid borne [121]. *Arthrobacter* sp. RC100 harbors three plasmids (pRC1, pRC2, and pRC300) of which conjugative plasmids, pRC1 and pRC2, encode enzymes for Carbaryl degradation up to gentisic acid. Enzymes involved in the conversion of gentisic acid to central carbon metabolites are encoded by genes located on the chromosome [115]. A *Rhizobium* sp. strain AC100, which transforms Carbaryl to 1-naphthol, harbors a plasmid pAC200 carrying gene *cehA* encoding CH as a part of Tn*ceh* transposon flanked by insertion element-like sequence (*ist*A and *istB*, Figure 4). *ist*A and *istB,* which comprise the ISR*sp3* element, showed homology to IS*1600* element from *A. eutrophus* NH9 and of IS*1326* from *P. aeruginosa* of the IS*21* family. Treating cells with plasmid curing agents like mitomycin-C resulted in deletion of *cehA* from the Tn*ceh* leaving behind the insertion elements as a part of plasmid pAC200d due to homologous recombination among ISR*sp3* [116]. This depicts the instability of the system and loss of catabolic genes by the activation of IS elements. *Sphingomonas* sp. strain CF06, which degrades carbofuran and its derivatives (including Carbaryl), harbors five plasmids- pCF01, pCF02, pCF03, pCF04, and pCF05. Plasmid curing and conjugation experiments revealed that the degradation property was associated with plasmid(s). Southern hybridization studies revealed a high degree of sequence similarity among pCF01 and pCF02, and pCF03 and pCF04, indicating gene duplication, which could be due to lack of positive regulatory system. Various IS elements were found to be present on pCF01, pCF02 and pCF03. The presence of active IS elements as well as gene duplications indicate that the system is in an early stage of evolution [67]. In Carbaryl degrading consortia of two *Pseudomonas* spp., the strain 50581 harbors a conjugative plasmid, pCD1 (50 kb) which encodes for CH, while chromosomally located genes from strain 50552 encode enzymes for 1-naphthol degradation [43].

**Figure 5:**
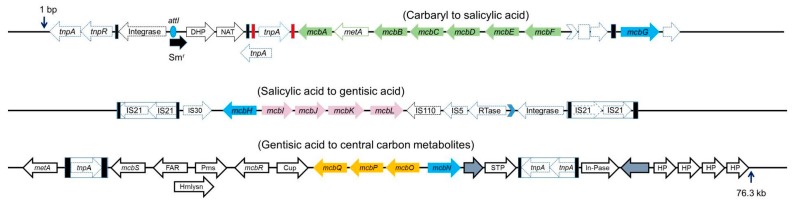
The organization of genes encoding enzymes responsible for Carbaryl degradation as three distinct transposons in *Pseudomonas* sp. strain C5pp. Genes are located on 76.3 kb long Supercontig-A (Accession number: KU522233.1). Inverted repeats (IRs) are depicted in red and black boxes. Arrow direction indicates the probable transcription direction. Blue color arrows depict genes encoding the probable regulators. Figure is not to the scale. Genes and protein encoded are transposase (*tnpA* and *tnpR*); integrase; *attI* site; streptomycin resistance (*Sm^r^*); dihydropterate synthetase (DHP); N-acetyl transferase (NAT); *mcbA*, Carbaryl hydrolase; *metA*, conserved protein; *mcbB,* 1,2-dihydroxynaphthalene dioxygenase; *mcbC*, 1-naphthol 2-hydoxylase; *mcbD*, 2-hydroxychromene 2-carboxylate isomerase; *mcbE*, *trans*-*o*-hydroxybenzylidene pyruvate hydratase-aldolase; *mcbF*, salicylaldehyde dehydrogenase; *mcbG*, LysR family regulator flanked by truncated transposons (depicted as dotted lines). IS21 insertion elements with inverted repeats; IS30 insertion element; *mcbH*, transcriptional regulator NahR; *mcbI*, ferredoxin reductase; *mcbJ*, salicylate 5-hydroxylase large oxygenase component; *mcbK*, salicylate 5-hydroxylase small oxygenase component; *mcbL*, ferredoxin; other transposase and insertion elements present downstream of the transposon. *metA*, conserved protein; *tnpA,* transposase; *mcbS*, TetR family transcriptional regulator; FAR, fusaric acid resistance; Hmlysn, hemolysin; Pms, permease; *mcbR*, LysR family transcriptional regulator; Cup, pirin and cupin2 superfamily related protein; *mcbQ*, maleylpyruvate isomerase; *mcbP*, fumaryl pyruvate hydrolase; *mcbO*, gentisate dioxygenase; *mcbN*, LysR family transcription regulator; transposase; STP, serine/threonine phosphatase; *tnpA,* transposase; In-Pase, inositol phosphatase; recombinase; HP, hypothetical protein.

Soil isolates *Pseudomonas* spp. strains C4, C5pp and C6 utilizes Carbaryl via salicylic acid and gentisic acid (Figure 2, [30]). Based on metabolic studies like enzyme induction, cell respiration and biotransformation, the Carbaryl degradation pathway was hypothesized to be divided into *upper*, *middle* and *lower* segments; and genes are probably arranged as three distinct operons induced by respective carbon source [122]. The degradation property was found to be very stable and could not be cured by nutritional conditions or chemical agents. Southern hybridization analyses indicate genes encoding degradative enzymes are located on the host genome [122]. The draft genome sequence of *Pseudomonas* sp. C5pp, primer walking, and gap filling PCR studies yielded a 76.3 kb sequence, referred to as Supercontig-A, harboring all genes required for Carbaryl degradation (Figure 5), [123,124]. Genes encoding enzymes responsible for the conversion of Carbaryl to salicylic acid (*upper* operon), salicylic acid to gentisic acid (*middle* operon), and gentisic acid to central carbon metabolites (*lower* operon) are probably under the regulation of transcription regulators encoded by *mcbG, mcbH* and *mcbN*, respectively (Figure 5).

The draft genome (6.15 Mb) analysis revealed the presence of 42 MGEs and 36 GEIs, out of which 17 MGEs were located in Supercontig-A (76.3 kb), indicating this region could be a hotspot for genome alterations [123,124]. The *upper* and *middle* operon region showed lower G+C content (54%) compared to the genome (62.65%) of strain C5pp. Whereas, the *lower* operon region showed comparable (60%) G+C content. This skewing of G+C content suggests that genes of the *upper* and *middle* pathways probably have a different ancestral origin and have been acquired through HGT events and assembled in proximity to each other. This is evident from the fact that 40% of MGEs are located in the Supercontig A (which is 1% of the total genome) [123,124]. Of the 36 GEIs, 3 were found to be a part of Supercontig-A. The *upper* pathway genes, except the regulator *mcbG* constitute a single GEI (13.8 kb), whereas the *middle* (6.5 kb) and the *lower* (9.6 kb) pathway genes, including their respective regulators, were established to be parts of two distinct GEIs (Figure 5). Interestingly, *P. putida* XWY-1, isolated from waste water in Shangong, China, 4750 km away from the site of isolation of the strain C5pp (Mumbai, India), showed a similar gene arrangement and nucleotide sequence for the genes involved in Carbaryl mineralization; however, these genes were reported to be plasmid borne [120].

In *Pseudomonas* sp. strain C5pp, the upstream region of the *upper* pathway genes showed class-I integron features, which showed high identity (95–99%) with Tn*6217* [124]. Other features present were transposase, 25 bp left-end repeat (92% homology to IR_i_), *attI* site, 5′ and 3′ conserved segment, resistance to streptomycin and two additional genes. The regulator *mcbG* is flanked by truncated transposases, which showed a similarity to ISP*a20* of IS3 family at the left end and ISP*st7* of IS5 family at the right end. The presence of partial transposase sequences indicate that their function has probably been lost due to decay linked recombination events and these might be the leftover sequences of previously functional ICE. The region consisting of *middle* pathway genes, *mcbIJKL*, flanked by IS21 family insertion repeats, exhibits class-I composite transposon like features and is referred to as the catabolic transposon Tn*C5ppsal* [123]. Members belonging to class-I composite transposon have been known to harbor gene clusters involved in degradation of xenobiotics [25]. The transposases present at the 3’ end of the cluster show a high degree of similarity to the IS110 and IS5 family, reverse transcriptase, group-II intron D1-D4-2, and leucine-zipper class of integrase. As depicted in Figure 5, the insertion elements of the IS21 family are present in inverted orientation, which could be the reason for the stability of the catabolic transposon in the genome of strain C5pp. Also, it has been reported that the increase in size of the intervening sequence of the composite transposon lowers the transposition frequency [123,124]. The lower pathway genes encoding for catabolism of gentisic acid to central carbon metabolites, are hypothesized to be a part of class-I composite transposon, which is bordered by non-identical insertion elements showing sequence similarity with ISP*a1635* and IS*481* [123,124]. In addition to this, the prediction of the probable ancestral origin of the genes using the IMG-JGI server indicates that *mcbBC*, *mcbEF*, and *mcbIJKL* were probably derived from beta-proteobacteria; *mcbA* and *mcbM* from alpha-proteobacteria; and *mcbD, mcbGH* and *mcbNOPQ* from gamma-proteobacteria.

The steps involved in the conversion of 1,2-dihydroxynaphthalene to salicylate appears to be common in both naphthalene and Carbaryl degradation pathways. Pair-wise sequence alignment (nucleotide as well as amino acid) of genes *nahC, nahD, nahE* and *nahF* (encoding these metabolic steps) from various naphthalene degrading Pseudomonads (Figure 3) and genes *mcbB*, *mcbD*, *mcbE*, and *mcbF* from Carbaryl degrading *Pseudomonas* sp. C5pp (Figure 5) revealed higher (~90–100%) identity amongst 2-hydroxybenzylidene pyruvate hydratase-aldolase as well as salicylaldehyde dehydrogenase encoding genes while 2-hydroxychromene 2-carboxylate isomerase showed moderate (~44–52%) identity. The phylogenetic analysis of Carbaryl degradation genes has been described in detail by Trivedi et al. [123,124]. Interestingly, 1,2-Dihydroxynaphthalene dioxygenase (12DHNDO) from strain C5pp, which belongs to class-II extradiol dioxygenases displayed low (4–10%) identity with 12DHNDOs from naphthalene degraders (class-I) as compared to protocatechuate 3,4-dioxygenase from lignin degraders *Pseudomonas humi* (74%) and *Burkholderia* sp. LIG30 (73%) [123,124]. Among various naphthalene degraders, irrespective of location, these genes displayed high (86–98%) identity. In strain C5pp, genes encoding CH and 1NH were hypothesized to be acquired from other degraders. Phylogenetic analysis revealed that CH from C5pp belongs to a new family of esterase with new conserved motif [124]. Enzyme 1NH from strain C5pp, which showed 47% activity on 2,4-dichlorophenol as substrate, yielded 55% identity with 2,4-dichlorophenol 6-monoxygenase from *Paraburkholderia zhejiangensis* [123,124]. This suggests that the strain has probably acquired the relevant gene, followed by mutations to evolve the enzyme, which will now accept 1-naphthol as its primary substrate. All of these observations indicate that the Carbaryl degradation genes must have been acquired through HGT followed by multiple transposition events leading to integration into the genome, giving a stable degradation phenotype in *Pseudomonas* sp. strain C5pp even in the absence of selection pressure.

Besides HGT events and assembly of genes in the form of operons, *Pseudomonas* sp. C5pp shows cellular level adaptation by compartmentalization of enzymes to tackle the toxicity of 1-naphthol [125,126]. This adaptation helps in degradation of higher concentration of Carbaryl (1% tested so far). The ability to metabolize Carbaryl at high concentration can be attributed to the presence of a low affinity CH (K*_m_*, 100 μM) in the periplasm of the bacterium [126]. This enzyme catalyses the conversion of Carbaryl to 1-naphthol (more toxic and recalcitrant than Carbaryl) in the periplasm, minimizing the interaction of 1-naphthol with cytoplasmic components. 1-Naphthol, transported across the membrane through diffusion and partition processes into the cytoplasm, is ring-hydroxylated by high affinity cytosolic 1NH (K*_m_*, 10μM) to 1,2-dihydroxynaphthalene and subsequently metabolized to central carbon pathway intermediates. The compartmentalization of enzymes involved in the degradation suggests a successful strategy evolved by *Pseudomonas* sp. strain C5pp for efficient degradation of Carbaryl at high concentration. 

## 6. Conclusions

Bacteria, which are present ubiquitously in nature, with a simple unicellular structure and ability to grow faster, have made their niches very dynamic, metabolically as well as genotypically plastic, and hence hot spots for the evolution of new trait(s). During the process of adaptation to strenuous conditions, microbes might acquire new genes and functions to enhance survivability. In addition, they might mutate already existing and/or acquired genes. The former approach is mediated through MGEs, which are the major players responsible for genome plasticity. Microbes evolve genes for novel pathways encoding xenobiotic degradation by the above mechanisms, which are further assembled as transcription unit(s)/operon(s), so as to finetune their regulation. Thus, only the required enzymes of the degradative pathway or segment of the pathway are activated by the respective carbon source. Further, the mutations in the acquired gene(s), shuffling/rearrangement of genes and compartmentalization of metabolic steps grant an additional advantage to the organism to adapt and utilize the compound more efficiently, as observed in the case of Carbaryl metabolism by *Pseudomonas* sp. C5pp. Apart from plasmids and transposons, the genes for aromatic degradation have also been found to be present as a part of GEIs/ICEs in the genome of various organisms including *Pseudomonas*. Integration of the degradation property into the host genome may impart additional stability to the phenotype, as generally the plasmids are lost in the absence of selection pressure. Therefore, by employing various strategies, microbes display an ability to adapt to challenging environments.

## Figures and Tables

**Table 1:** Naphthalene and Carbaryl degradation pathways and location of genes from various isolates.

Organism	Pathway, ring-cleavage mode	Chromosome/plasmid (kb); Operon size (kb)	References
***A. Naphthalene degradation***			
*Pseudomonas putida* G7	Catechol, *meta*	Plasmid, NAH7 (82); *nah* (10) and *sal* (8)	[99]
*Pseudomonas putida* strain NCIB 9816-4	Catechol, *meta*	Plasmid, pDTG1 (88); *nah* (9.5) and *sal* (13.4)	[102]
*Pseudomonas* sp. strain ND6	Catechol, *meta*	Plasmid, pND6-1 (102); *nah* (10) and *sal* (18)	[109]
*Pseudomonas fluorescens* strain PC20	Catechol, *meta*	Plasmid, pNAH20 (88); *nah* (6) and *sal* (13)	[110]
*Pseudomonas putida* strain AK5	Gentisic acid	Plasmid, pAK5 (-); *nah* (6.7) and *sal* (12.2)	[103]
*Pseudomonas* sp. AS1	Catechol, *ortho*	Plasmid, pAS1 (82)	[111]
*Pseudomonas putida* strain PMD-1	Catechol, *meta*	Chromosome and Plasmid, pMWD-1	[104]
*Pseudomonas stutzeri* AN10	Catechol, *meta*	Chromosome, *nah* (11.5) and *sal* (16)	[105,106]
*Pseudomonas putida* CSV86	Catechol, *meta*	ICE, *nah* (8.2) and *sal* (9.8)	[91]
***B. Carbaryl degradation***			
*Achromobacter* sp.	Hydroquinone, Catechol,	- *	[112]
*Pseudomonas* sp. NCIB 12043	Gentisic acid	-	[113]
*Pseudomonas* sp. NCIB 12042	Catechol, *meta*	-	[113]
*Rhodococcus* sp. NCIB 12038	Gentisic acid	-	[113]
Consortia *Pseudomonas* spp. isolate 50581 and 50552	Catechol	Plasmid, pCD1 (50) in isolate 50581 encodes Carbaryl hydrolase; Chromosome encodes degradative enzymes for 1-naphthol in isolate 50552	[43]
*Blastobacter* sp. strain M501	Hydrolysis to 1-naphthol	-	[114]
*Sphingomonas* sp. strain CF06	Gentisic acid	Plasmids pCF01, pCF02, pCF03, pCF04, and pCF05, role of each plasmid is not clear	[67]
*Arthrobacter* sp. RC100	Gentisic acid	Plasmid, pRC1 (110) encodes Carbaryl hydrolase; Plasmid, pRC2 (120) encodes enzymes for 1-naphthol to gentisic acid; chromosome encodes enzymes for utilization of gentisic acid	[115]
*Rhizobium* sp. strain AC100	Partial hydrolysis to 1-naphthol	Plasmid, pAC200 encodes Carbaryl hydrolase	[116]
*Micrococcus* sp.	Gentisic acid	-	[117]
*Pseudomonas* sp. strain C4	Gentisic acid	Chromosome	[30]
*Pseudomonas* sp. strain C5	Gentisic acid	Chromosome	[30]
*Pseudomonas* sp. strain C6	Gentisic acid	Chromosome	[30]
*Burkholderia* sp. C3	Catechol and Gentisic acid	-	[118]
*Pseudomonas* sp. strain C7	Gentisic acid	-	[119]
*Pseudomonas putida* XWY-1	Gentisic acid	Plasmid, pXWY (400) encoding all enzymes of Carbaryl degradation	[120]

Note: *: -, Not known/reported
